# Lower Extremity Open Fractures Fix and Flap: Does Initial Management in Non-specialized Hospitals Really Compromise its Outcome?

**DOI:** 10.7759/cureus.60380

**Published:** 2024-05-15

**Authors:** Henrik Lauer, Benedetta Vasselli, Michael Bressler, Claudius Illg, Heiko Baumgartner, Benedikt Johannes Braun, Johannes Heinzel, Jonas Kolbenschlag, Adrien Daigeler, Johannes Tobias Thiel

**Affiliations:** 1 Department of Hand-Plastic, Reconstructive and Burn Surgery, Berufs-Genossenschaft Klinik Tuebingen, University of Tuebingen, Tuebingen, DEU; 2 Department of Trauma and Reconstructive Surgery, Berufs-Genossenschaft Klinik Tuebingen, University of Tuebingen, Tuebingen, DEU

**Keywords:** gustilo-anderson classification, lower extremity, godina, free tissue transfer, open fractures

## Abstract

Introduction

Managing open lower extremity fractures is challenging, with potential complications such as amputation and infection. The aim of the study was to determine whether the time delay and initial treatment of the patients treated in a non-specialized hospital before being transferred to a dedicated level I trauma center led to a worse outcome.

Methods

Retrospective data from 44 patients (37 males and seven females) undergoing free tissue transfer for lower extremity open fractures from January 2017 to December 2022 were analyzed. Group A received primary care externally and was later transferred for definitive treatment (n=17, 38.6%), while group B received initial care at a level I trauma center (n=27, 61.4%). Surgical outcomes, complications, the duration of the hospital stay, and assessment times were compared. Various demographic variables, co-morbidities, prior interventions, and flap types were analyzed.

Results

Average age (A: 55.1±16.7; B: 38.7±19.8 years; p=0.041), overall hospitalization (A: 55.7±22.8; B: 42.8±21.3 days; p=0.041), and time to soft tissue reconstruction differed significantly between groups (A: 30.7±12.2; B: 18.9±9.3 days; p=0.013). Overall, 31.8% had multiple injuries without statistical differences between groups A and B (29.4% vs. 33.3%; p>0.05). There were no statistical differences between the groups in terms of major and minor complications and bone healing characteristics. Limb salvage was successful overall in 93.2% (A: 94.1%; B: 92.6%; P>0.05). Major complications occurred in 9.1%; three patients underwent major amputation (A: n=2; B: n=1). Minor complications were observed in 43.2% of patients (partial flap necrosis, wound dehiscence and non-union; A: 41.2%; B: 44.4%; p>0.05). Overall, 65.9% of patients (A: 64.7%; B: 66.7%; p>0.05) experienced uneventful bone healing, while 18.2% of patients (A: 23.5%; B: 14.8%; p>0.05) experienced delayed healing. Flaps used were mostly musculocutaneous (71.7%). Various assessed demographic characteristics, including age and presence of polytrauma, showed no significant influence on complications (p>0.05).

Conclusion

Although there is a significant difference in the time course of externally treated patients with open fractures, prolonged treatment is not associated with a higher complication rate or compromised bone healing outcome. Despite the findings, it is important to avoid delays and strive for interdisciplinary collaboration.

## Introduction

The treatment of open and complex fractures of the lower extremity combined with soft tissue damage remains a challenge due to the risk of osteosynthesis failure, infection, and amputation [1]. Prompt and appropriate management is of utmost importance to avoid complications and optimize patient outcomes [2,3]. Traditionally, patients with such injuries have been referred to specialized tertiary centers for definitive care, combining orthopedic and plastic as well as vascular surgery, a concept known as “orthoplastic surgery” [4]. However, the primary transfer of a significant portion of these patients to district general hospitals without access to such facilities results in a delay in the process and definitive surgical treatment [5].

The aim of this retrospective study was to evaluate whether patients with open fractures of the lower extremity and defects requiring flap surgery, who received primary care in non-specialized healthcare facilities prior to transfer for definitive osteosynthesis and soft tissue reconstruction, exhibit comparable outcomes following surgery. By assessing the outcomes between patients managed in non-specialized hospitals and those treated primarily in specialized centers, we aimed to gather valuable insights into the ongoing discourse in trauma and orthopedic care. Furthermore, understanding the impact of initial care settings on patient outcomes can positively influence healthcare policy and resource allocation.

## Materials and methods

In this analysis, retrospective data from 44 patients (37 (84.1%) males and 7 (15.9%) females) was included, who underwent free tissue transfer due to open fractures of the lower extremity in a level I trauma center between January 2017 and December 2022. Based on the Gustilo and Anderson classification, only grades IIIB (n=39, 88.6%) and IIIC (n=5, 11.4%) injuries were included [6].

Patients were categorized into two groups: Group A consisted of individuals who received primary care at external healthcare facilities and were subsequently transferred to our institution for definitive treatment. Group B is comprised of patients who initially received primary care at our hospital. Surgical outcomes, including (1) fracture healing, (2) the need for revision surgeries due to minor complications such as wound healing disorders, partial flap necrosis, surgical site infections and non-unions, and (3) major complications such as amputations, were evaluated. Osteomyelitis was distinct from surgical site infections and was also considered a major complication. Moreover, the total duration of the hospital stays, the time from injury to soft tissue reconstruction, and the time to plastic reconstructive assessment were documented and compared. X-ray and CT examinations were used to assess bone healing. If the bone healed within the first three months after osteosynthesis, it was assumed to have healed in a timely fashion. Completed bone healing between three and six months postoperatively was assessed as “delayed bone healing." Failed bone healing beyond six months was assessed as a non-union [7,8]. In addition, the presence of the additional variables: (1) vascular damage, (2) compartment syndrome, and (3) polytrauma were compared group-dependently for their correlation with fracture healing, the occurrence of minor and major complications, and prolonged hospital stays.

To ensure comprehensive data collection, various demographic variables, including age, sex, co-morbidities, and prior therapeutic interventions, were analyzed. The types of free flaps (muscular versus fasciocutaneous) were recorded as well. 

We included all patients with open fractures in the lower extremity (tibia and fibula) with extensive soft tissue damage (Gustilo-Anderson IIIb and IIIc) who underwent free tissue transfer for wound closing. Ethical approval was obtained from the local ethics committee (Project number: 333/2023BO2).

Data analysis

All statistical analyses were performed with MATLAB for Windows (Version R2021a, MathWorks, Natick, MA, USA) with a significance level of p<0.05. All data were tested for normal distribution using the Kolmogorov-Smirnov test. Due to the small sample sizes, nonparametric tests were used for all analyses.

To test the effects of the groups (A, B) and the presence of additional factors such as vascular damage and no vascular damage for significance regarding temporal data (1) “stay after flap," (2) "days from injury to surgery," (3) "days from presentation to reconstruction," (4) "total hospital stay," (5) "follow-up months," and regarding (6) “frequency of vacuum therapy," the Wilcoxon rank-sum test was used. 

To test the effects of the groups (A, B) and the presence of additional factors such as (1) vascular damage and no vascular damage, (2) polytrauma and no polytrauma, (3) compartment and no compartment regarding frequency data ("major complications," "minor complications," "flap revision," and “bone healing on time," "delayed bone healing," "non-union," and "amputation"), the chi-square test was used.

To assess the effects of the factors age (1), "total days of hospital stay" (2), "days from injury to surgery" (3), "days from representation to reconstruction" (4), "follow-up months" (5), and "use of vacuum therapy" (6), all subjects were categorized into groups and tested for significance using the chi-square test with the p-level adjusted by Bonferroni correction.

To assess effects of age, subjects were categorized into six groups (years: <25, 25-34, 35-44, 45-54, 55-64, 65+); for “total days of hospital stay," subjects were categorized into five groups (days: <20, 20-39, 40-59, 60-79, 80+); for “days from injury to surgery," subjects were categorized into six groups (days: <10, 10-19, 20-29, 30-39 40-49, 50+); for “days from representation to reconstruction," subjects were categorized into six groups (days: <5, 5-9, 10-14, 15-19, 20-24, 25+); for “follow-up months," subjects were categorized into five groups (months: <10, 10-19, 20-29, 30-39, 40+); for “change of vacuum dressing," subjects were categorized into six groups (times: 0, 1, 2, 3, 4, 5+, Table 1).

**Table 1:** Categorizing groups for assessment of "age", "total days of hospital stay," “days from injury to surgery," “days from representation to reconstruction," “follow-up months" and "use of vacuum therapy." All subjects were categorized into groups and tested for significance using the chi-square test with the p-level adjusted by Bonferroni correction.

Assessment effects of "age"	Years	Assessment effects of "total days of hospital stay"	Days	Assessment effects of "days from injury to surgery"	Days	Assessment effects of "days from representation to reconstruction"	Days	Assessment effects of "follow-up months"	months	Assessment effects of "change of vacuum dressing"	Times
I	<25	I	<20	I	<10	I	<5	I	<10	I	0
II	25-34	II	20-39	II	10-19	II	5-9	II	10-19	II	1
III	35-44	III	40-59	III	20-29	III	10-14	III	20-29	III	2
IV	45-54	IV	60-79	IV	30-39	IV	15-19	IV	30-39	IV	3
V	55-64	V	>79	V	40-49	V	20-24	V	>39	V	4
VI	>64			VI	>49	VI	>24			VI	>4

## Results

A total of 44 patients were included in the study, comprising 37 (84.1%) males and 7 (15.9%) females. The distribution among the groups was as follows: 17 patients were pre-treated externally in other hospitals (group A, 38.6%), and 27 patients were treated without prior treatment elsewhere (group B, 61.4%). Primary care was provided in all cases, whether external or internal primary treatment, in less than six hours after the accident. 

The average age was 45.1±19.9 years (A: 55.1±16.7; B: 38.7±19.8; p=0.0069). The overall length of hospital stay was 47.8±22.3 days (A: 55.7±22.8; B: 42.8±21.3; p=0.041). The time interval from injury to soft tissue reconstruction averaged 23.5±11.8 days and differed also significantly between the two groups (A: 30.7±12.2; B: 18.9±9.3; p=0.0022). 

The postoperative hospital stay following flap reconstruction was 29.8 ±18.8 days and showed no significant differences between the two groups (A: 29.2±16.2; B: 30.1±20.9; p>0.05). Prior to flap reconstruction, a vacuum dressing was applied and changed in combination with debridement on average 2.5±1.7 times before final tissue reconstruction (A: 2.7±2.1; B: 2.3±1.5; p>0.05). The follow-up period extended to an average of 21.4±14 months (A: 20.1±14.1; B: 21.5±14.6; p>0.05). 

Among the 44 patients, 14 (31.8%) experienced polytrauma (A: n=5 (11.4%), B: n=9 (20.5%); p>0.05). Additionally, seven patients (15.9%) received supplementary treatment due to compartment syndrome (A: n=2 (4.5%), B: n=5 (11.4%); p>0.05). Five patients (11.4%) required surgical intervention for critical vascular injuries resulting from open fractures. All these patients belonged to group B (A: n=0, B: n=5 (11.4%); p>0.05). 

Limb salvage was successfully performed in 41 patients (93.2%). Major complications were observed in four patients (9.1%; A: n=1 (2.3%), B: n=6 (13.6%); p>0.05). Of these, three patients underwent major amputation during treatment (6.8%, A: n=1 (2.3%), B: n=2 (4.5%); p>0.05). One patient received continued treatment due to osteomyelitis (2.3%, group B). 

Minor complications occurred in 19 patients (43.8%, A: n=7 (15.9%), B: n=12 (27.3%); p>0.05). Six of them exhibited non-union (13.6%, A: n=1 (2.3%), B=5 (11.4%); p>0.05), necessitating further surgical procedures. One of these patients with non-union received major amputation (2.3%, also counted as a major complication, group B). Four of the patients demonstrated bony revascularization in the further course of treatment (9.1%; all of them belonged to group B). The other minor complications were partial flap necrosis (A: n=2 (4.5%); B: n=4 (9.1%); p>0.05); wound dehiscence and surgical site infection (A: n=4 (9.1%); B: n=2 (4.5%); p>0.05); and hematoma formation requiring revision in the area of the donor site (A: n=0; B: n=1 (2.3%); p>0.05). All patients were discharged for outpatient follow-up with closed and non-irritated wound conditions.

**Figure 1:**
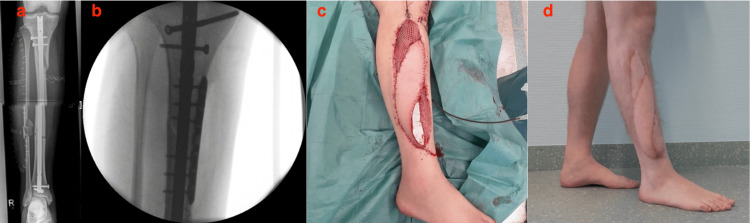
Definitive osteosynthesis during flap surgery. Primary internal bony fixation was done externally (a). (b) and (c) show the corrective osteosynthesis and the treatment using the latissimus dorsi flap. Image (d) shows the healing status 15 months after the operation.

Twenty-six patients were treated with definitive osteosynthesis care during flap surgery (59.1%, A: n=8 (18.2%), B: n=18 (40.9%); p>0.05), while 17 patients received definitive osteosynthesis before flap surgery (38.6%, A: n=9 (20.5%), B: n=8 (18.2%); p>0.05; Figure 1, Tables 2, 3).

**Table 2:** Age and treatment characteristics. The data has been represented as mean±SD. P-value is considered significant as p<0.05.

	Age (years)	Overall length of hospital stay (days)	Time interval from injury to soft tissue reconstruction (days)	Postoperative hospital stay (days)	Change of vacuum dressing (N)	Definitive osteosynthesis care during flap surgery (N)	Definitive osteosynthesis before flap surgery (N)	Definitive osteosynthesis after flap surgery (N)	Muscular flaps for wound closure (N)	Adipocutaneous flaps for wound closure (N)
Total	45.1±19.9	47.8±22.3	23.5±11.8	29.8±18.8	2.5±1.7	26 (59.1%)	17 (38.6%)	1 (2.3%)	32 (72.7%)	13 (29.5%)
A	55.1±16.7	55.7±22.8	30.7±12.2	29.2±16.2	2.7±2.1	8 (18.2%)	9 (20.5%)	0	12 (27.3%)	5 (11.4%)
B	38.7±19.8	42.8±21.3	18.9±9.3	30.1±20.9	2.3±1.5	18 (40.9%)	8 (18.2%)	1 (2.3%)	20 (45.5%)	8 (18.2%)
	p=0.0069	p=0.0414	p=0.0022	p>0.05	p>0.05	p>0.05	p>0.05	p>0.05	p>0.05	p>0.05

**Table 3:** Overview of associated injuries, treatment times of osteosynthetic care, and complication rates. *Wound healing disorders, partial flap necrosis, surgical site infections and non-unions. The data has been represented as mean±SD. The P-value is considered significant as p<0.05.

	N	Polytrauma	Compartment syndrome	Vascular damage	Definitive osteosynthetic care before flap	Definitive osteosynthetic care during flap	Definitive osteosynthetic care after flap	Flap revision	Bone healing on time	Delayed bone healing	Minor complications*	Non-union	Amputation	Osteomyelitis
Total	44	14 (31.8%)	11 (25%)	5 (11.4%)	17 (38.6%)	26 (59.1%)	1 (2.3%)	3 (6.8%)	29 (65.9%)	8 (18.2%)	19 (43.2%)	6 (13.6%)	3 (6.8%)	1 (2.3%)
A	17 (38.6%)	5 (11.4 %)	2 (4.5%)	0	9 (20.5%)	8 (18.2%)	0	1 (2.3%)	11 (25%)	4 (9.1%)	7 (15.9%)	1 (2.3%)	1 (2.3%)	0
B	27 (61.4%)	9 (20.5%)	9 (20.5%)	5 (11.4%)	8 (18.2%)	18 (40.9%)	1 (2.3%)	2 (4.5%)	18 (40.9%)	4 (9.1%)	12 (27.3%)	5 (11.4%)	2 (4.5%)	1 (2.3%)
		p>0.05	p>0.05	p>0.05	p>0.05	p>0.05	p>0.05	p>0.05	p>0.05	p>0.05	p>0.05	p>0.05	p>0.05	p>0.05

**Figure 2:**
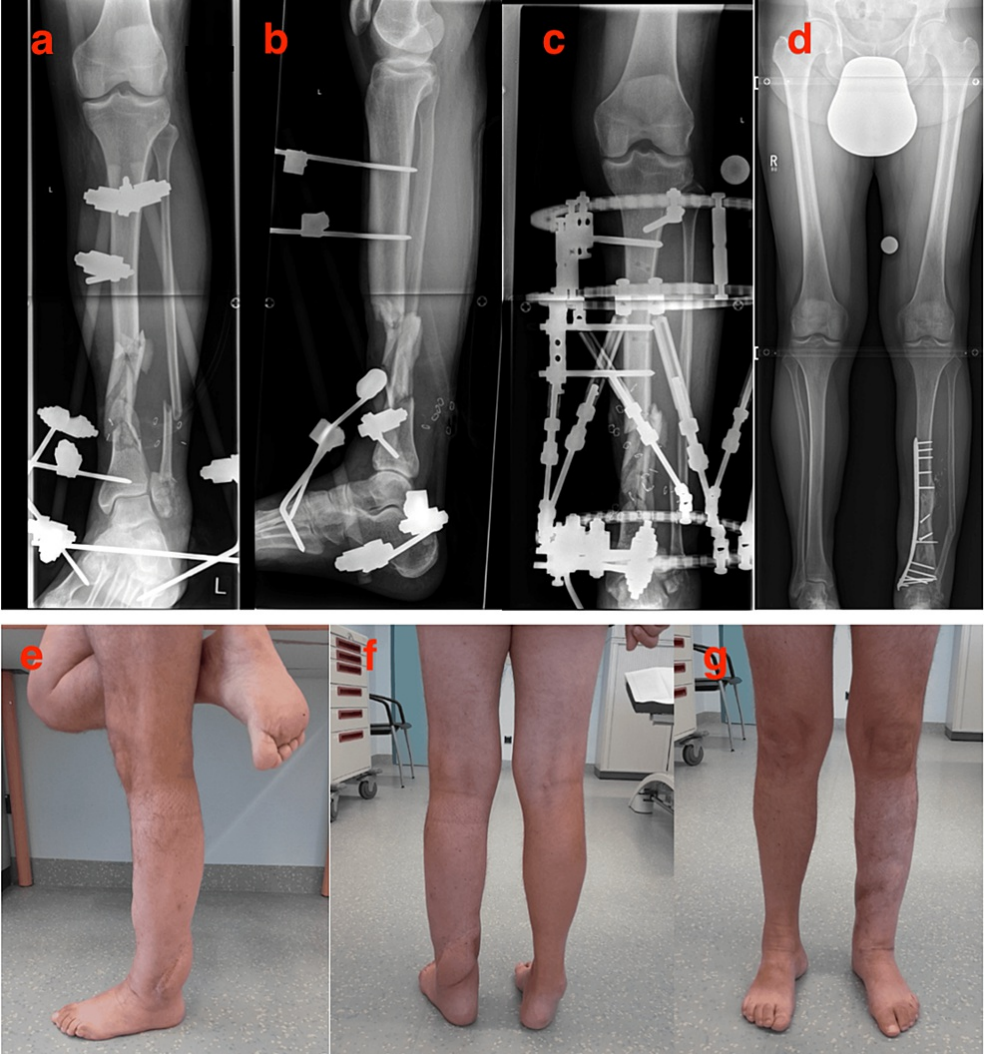
Definitive osteosynthesis after flap surgery. On the day of the accident, wound debridement and the placement of an external fixator were carried out (a) and (b), spanning the ankle joint. After a stabilized wound situation, a Hexapod system ring fixator was applied (c), and defect coverage was carried out using a free latissimus dorsi flap. Eleven months after dynamic axis correction, bone edges were refreshed, spongiosa graft and bone graft were taken from the iliac crest, and the iliac crest graft was fitted, followed by definitive plate osteosynthesis (d). The lower images depict the clinical condition 23 months after the accident (e)-(g).

One patient (group B) received definitive osteosyntheses after flap surgery (Figure 2, Table 3).

Bone healing characteristics

In 29 cases, the patients displayed timely bone healing (65.9%, A: n=11 (25%), B: n=18 (40.9%); p>0.05). Eight patients showed delayed bone healing (18.2%, A: n=4 (9.1%), B: n=4 (9.1%); p>0.05). In the statistical analysis, there was no evidence of a significant difference for regular bone healing, the occurrence of delayed bone healing or the occurrence of non-union in the comparison of groups A and B (p>0.05). The time of definitive osteosynthesis also showed no significant influence on the complication rate (p>0.05). 

Flap characteristics

Most frequently, muscular flaps were employed for wound closure (72.7%, A: n=12 (27.3%), B: n=20; p>0.05). Specifically, the latissimus dorsi flap was utilized 19 times (43.2%), followed by the gracilis flap, which was used in 13 cases (29.5%). The anterolateral thigh flap (ALT) was selected in 12 instances (27.3%). Additionally, the parascapular flap was performed once (2.3%). In one case, two flap reconstructions were required for wound closure (group B). Flap revision occurred in three cases (6.8%, A: n=1 (2.3%), B: n=2 (4.5%); p>0.05). Complete flap loss did not occur in any patient. The type of flap (muscular versus fasciocutaneous) showed no significant differences in terms of minor complications, major complications, bone healing characteristics, and length of hospital stay (p>0.05).

Other characteristics

Age, hospitalization, and the time between trauma and flap surgery had no significant influence on minor or major complications (p>0.05). The time of definitive osteosynthesis in relation to flap coverage revealed no differences (p>0.05). The presence of vascular damage also had no influence on the complication rate. No correlation with a prolonged hospital stay was found here either (p>0.05). The presence of polytrauma had no effect on minor or major complications as well as on bone healing (p>0.05).

## Discussion

In the study presented, the results of patients who were pre-treated in non-specialized hospitals were compared with those who were initially treated in a level I trauma center following severe injuries of the lower extremity with the need for soft tissue reconstruction. 

Since the introduction of Godina's principles, the early reconstruction of massive tissue defects with free flaps was long regarded as best practice in the treatment of open fractures [2,3,9]. However, time management and scheduling of reconstruction are dependent on many factors, and patient status can be critical. The overall length of hospital stays and the time interval from injury to soft tissue reconstruction differed significantly in our two groups. There are many reasons why patient transfer is delayed, resulting in prolonged hospital treatment. In a meta-analysis that compared the outcomes following direct admission and early transfer to specialized trauma centers, the main reason for the delay in treating open tibia fractures included a shortage of beds in tertiary centers and delays in accessing the operating theater [5]. Other identified reasons were a delay in communication and a lack of experience [5,10]. Another reason could be the presence of life-threatening concomitant injuries since open fractures of the lower extremity very often occur due to high-speed trauma, which changes the treatment algorithm for open fractures [11]. Despite the presence of polytrauma in almost one-third of all patients, our patient population showed at least no significant differences in terms of the risk of minor or major complications as well as bone healing depending on whether the patient had been pre-treated externally or not. It can be assumed that patients pre-treated externally do not have to expect worse results.

The age was significantly different between the groups, with a notably higher average age in the group that received external pre-treatment. However, no influence on the outcome could be observed in this regard. Interestingly, a single-center, retrospective study with more than 1000 patients included showed that age influenced non-union risk in an unexpected way, with the highest rates observed in patients who were in the middle decades of their adulthood [12]. On the other hand, age itself had no influence on hospitalization, complication rate and bone healing characteristics in our study.

There were no significant differences in the choice of flap regarding minor and major complications and bone healing characteristics in our study as well. Muscular flaps were chosen in more than two-thirds of cases. The latissimus dorsi flap was chosen most frequently based on the operating surgeon’s familiarity with this flap and the specific anatomical and clinical considerations of each case. 

The choice of the free flap for soft tissue reconstruction is still a matter of debate [13]. Around the ankle, fasciocutaneous flaps could be more appropriate because skin grafts might be susceptible to minor trauma [14]. A retrospective review of soft tissue-free flaps used for traumatic foot and ankle defects showed that muscle flaps demonstrated higher rates of wound complications [15]. However, the outcomes of fasciocutaneous and muscle flaps have been shown to be comparable in managing lower extremity injury [16]. Another systematic review and meta-analysis including 2711 flaps revealed that there were also no significant differences in long-term post-operative outcomes but suggested that fasciocutaneous flaps should be preferred to avoid flap necrosis and donor-site complications. In our study, there was no association with total or partial flap necrosis, wound dehiscence and donor-site complications in regard to the choice of flap. 

An interesting question is whether the presence of vascular injuries influences the result of open fracture treatment. Based on the study by Chummun et al., the cases listed in our study with Gustilo type 3C injuries were patients with de-vascularized limbs [17]. Even though the risk of amputation appears to be significantly higher with previous vascular damage and interventions, the clientele of our patients (all belonged to group B) showed no correlation between vascular damage and complications rates (minor or major complications such as partial flap necrosis, total flap necrosis, amputation) [18]. However, with five patients in the overall collective, the number of cases with concomitant vascular damage can be classified as low. In addition, it must be noted that functional evaluation was not conducted. In cases involving avascular extremities, it is necessary to assume concomitant injuries to muscles and nerves, which can lead to a significantly poorer functional outcome [17]. 

Initial debridement and systematic antibiotic administration are essential for the further healing process and for the prevention of infection in open fractures [19-23]. Here, the complication rate regarding infections and bone healing characteristics between our groups of patients showed no significant difference. This suggests that once patients received the necessary surgical and medical interventions, subsequent management and outcomes were comparable regardless of the initial treatment facility. The number of debridement and changes of vacuum dressing did also not differ significantly between the groups (A: n=2.7; B: n=2.3). The use of negative pressure wound therapy (NPWT) prior to definitive surgical procedures to optimize wound conditions is discussed [24]. Evidence exists that NPWT leads to fewer infections in the acute trauma phase [25]. In contrast, a random control trial study showed that there is at least no benefit to the use of irrigation-pressure devices [26]. It must be said that NPWT must primarily be seen as a transitional method for wound coverage until final wound closure. However, it remains questionable to what extent it supports the treatment of open fractures [19].

The timing of wound closure is also discussed. As reported at the beginning, care within the first three days is a paradigm based on the study by Godina in 1986 [2]. Other studies have confirmed that early care within the first seven days minimizes the risk of infection [27,28]. In contrast, our results revealed that early care within the first seven days is unrealistic, especially when patients are transferred from external hospitals. We did not find that the risk of infection increases with longer treatment in the comparison of the two groups [28]. At 9.1%, the number of major complications in the two groups surveyed was higher than the 6.2% that resulted from early treatment within the first seven days in the systematically analyzed study by Pincus et al. [27]. This may very well be due to delayed care (in average 23.5 days), even if the 16.7% described by Pincus et al. for delayed coverage (>7 days) was not confirmed [27].

No question that delayed wound coverage can be carried out effectively, but prompt wound care should be sought to reduce complications [24]. In our cohort, however, there was no statistically significant difference in the case of external pre-treatment (A: 30.7 days; B: 18.9 days; p>0.05) and limb salvage was successful in the majority of cases (93.2%).

Our study revealed no differences in the timing of definitive osteosynthesis in relation to wound coverage and no differences in the timing of definitive osteosynthesis between the groups (p>0.05). From these findings, it can be inferred that the precise timing of definitive osteosynthesis concerning flap-plastic coverage may not be of paramount importance. Consistent with the observations of other researchers, we concur that aggressive debridement coupled with antibiotic therapy, along with a personalized transition from external fixation to definitive osteosynthesis, stands out as the pivotal element in the management of open fractures [29].

The study has several limitations that should be considered when interpreting its findings. Firstly, the use of retrospective, monocentric data introduces inherent biases and limits the generalizability of the results to a broader population. A lack of statistical significance does not necessarily mean there is no difference; it could indicate that the sample size may not be large enough to detect differences or that other influencing factors have not been adequately considered. Additionally, the study lacks detailed information on the specific type of fractures, such as distinguishing between a complicated tibial plateau fracture and an ankle dislocation fracture, which can significantly affect treatment outcomes. Furthermore, the absence of data on the functional outcomes of patients is a notable limitation. While the study assessed bone healing as satisfactory, it did not provide insights into the patient's functional status post-treatment, potentially overlooking persistent impairments resulting from the injury. Moreover, the study lacks information on patient-reported outcome measurements (PROMs), which are crucial for understanding the subjective experiences and perceptions of patients regarding their recovery and overall well-being. Higgin et al., for example, pointed out that patients suffering because of open tibial fractures have a significant risk of psychosocial harm, and those sustaining major polytrauma or amputation had the greatest risk of a poor outcome [30].

## Conclusions

In summary, this study provides insight into the outcomes of patients prehospitalized in county general hospitals compared to a level I trauma center. While there were significant differences observed in age and length of hospital stay, externally pre-treated patients showed equally good results in terms of bone healing and limb preservation. These findings should be interpreted in the context of the specific patient population and the clinical practices of the respective facilities. However, care should be optimized for the benefit of patients and treatment processes improved, especially because the complex nature of open fractures necessitates a multidisciplinary approach.
